# Irisin is an Effector Molecule in Exercise Rehabilitation Following Myocardial Infarction (Review)

**DOI:** 10.3389/fphys.2022.935772

**Published:** 2022-06-29

**Authors:** Shuguang Qin, Zhenjun Tian, Maxime Boidin, Benjamin J. R. Buckley, Dick H. J. Thijssen, Gregory Y. H. Lip

**Affiliations:** ^1^ Institute of Sports and Exercise Biology, School of Physical Education, Shaanxi Normal University, Xi’an, China; ^2^ Department of Cardiology, The Second Affiliated Hospital of Xi’an Jiaotong University, Xi’an, China; ^3^ Liverpool Centre for Cardiovascular Science, Liverpool John Moores University, Liverpool, United Kingdom; ^4^ Cardiovascular Prevention and Rehabilitation (EPIC) Center, Montreal Heart Institute, Montreal, QC, Canada; ^5^ School of Kinesiology and Exercise Science, Faculty of Medicine, Université de Montréal, Montreal, QC, Canada; ^6^ Liverpool Centre for Cardiovascular Science, University of Liverpool and Liverpool Heart and Chest Hospital, Liverpool, United Kingdom; ^7^ Cardiovascular and Metabolic Medicine, Institute of Life Course and Medical Sciences, University of Liverpool, Liverpool, United Kingdom; ^8^ Research Institute for Sport and Exercise Sciences, Liverpool John Moores University, Liverpool, United Kingdom

**Keywords:** irisin, exercise, myocardial infarction, cardiac rehabilitation, cardioprotection

## Abstract

**Background:** Regular exercise is an effective non-pharmacological therapy for treatment and prevention of cardiovascular disease (CVD). The therapeutic benefits of exercise are mediated partly through improved vascular and increase in metabolic health. Release of exercise-responsive myokines, including irisin, is associated with beneficial effects of exercise in CVD patients.

**Observations:** The present review provides an overview of the role of exercise in cardiac rehabilitation of patients with myocardial infarction (MI). Further, the role of irisin as a motion-responsive molecule in improving vascular and metabolic health is explored. Possible mechanism of cardioprotective effect of irisin-mediated exercise on myocardial infarction are also summarized in this review.

**Conclusion and significance of the review:** Irisin is associated with reduced inflammation, antioxidant properties, and anti-apoptotic effect, implying that it is a potential key mediator of the beneficial effects of exercise on vascular and metabolic health. The findings show that irisin is a promising therapeutic target for treatment of patients with cardiovascular disease, particularly post-MI. Further research should be conducted to elucidate the potential mechanisms of cardioprotective effects of irisin and explored whether irisin induced by exercise exerts rehabilitation effects post-MI.

## 1 Introduction

Exercise is an effective non-pharmacological intervention that improves cardiovascular health and function. Moreover, exercise alleviates cardiovascular disease (CVD) risk factors, and reduces all-cause mortality in CVD patients (Lear et al., 2017; Lin et al., 2020). Due to the dose-response relationship between exercise intensity and/or duration and overall cardiovascular benefit, the correct choice of exercise rehabilitation mode is particularly important for patients with CVD (Aengevaeren et al., 2017; Franklin et al., 2020). Exercise has been found to improve left ventricular function after myocardial infarction (MI) (Maessen et al., 2017; Alhumaid et al., 2022), which beneficial effects are partly mediated by promotion of cardiovascular function, mitochondrial biogenesis, and through stimulation of skeletal muscle to release myokines (Rosenkilde et al., 2018; Pinckard et al., 2019). However, its molecular mechanisms are not clear completely.

Irisin is mainly secreted in skeletal (Boström et al., 2012) and cardiac muscle tissue (Aydin et al., 2014), and is implicated in modulation of mitochondrial function and energy balance (Ouyang et al., 2020; Xin et al., 2020) lipid and glucose metabolism (Perakakis et al., 2017; Wang et al., 2020), and amelioration of impaired cardiac function in some metabolic disorders (Wang et al., 2017; Jiang et al., 2021). Previous findings show that circulating irisin level is lower in patients with chronic heart failure (Silvestrini et al., 2019), middle cerebral artery occlusion (MCAO) patients (Li et al., 2017), and subjects with Alzheimer disease (AD) (Kim et al., 2018) compared with normal subjects. Low irisin levels are associated with increase in levels of circulating inflammatory cytokines and/or angiotensin Ⅱ observed in various diseases. Exercise is the main inducer of irisin secretion both in healthy and dysregulated metabolism individuals (Huh et al., 2015). Studies have confirmed that exercise-induced irisin is correlated with improvement of cardiac function in general (Seo et al., 2020), which partly by modulating autophagy and mitochondrial function (Li et al., 2021a; He et al., 2021). Further previous animal studies report that irisin is implicated in cerebrovascular protective effects of exercise by alleviating ischemic neuron injury (Li et al., 2017; Lourenco et al., 2019). Irisin has a significant endothelial protective effect (determined by flow-mediated arterial dilation) (Wang et al., 2015) and inhibits progression of atherosclerosis (determined by flow-mediated arterial dilation (Lu et al., 2015; Chen et al., 2022). These findings indicate that irisin is a potential factor that mediates the protective effects of exercise in patients with cardio-cerebrovascular disease.

In the present review, cardioprotective effect of exercise on MI was explored. Moreover, the role of irisin, as an important exercise effector molecule in cardioprotective effects of exercise training was summarized. In addition, the possible cardiac protective mechanism of irisin reported in clinical studies and animal experiments was reviewed. The findings of the present review indicate that irisin plays an important role in exercise rehabilitation of MI.

## 2 Role of Exercise in Cardiac Rehabilitation of Patients With Acute Myocardial Infarction

### 2.1 Exercise-Based Cardiac Rehabilitation Conducted Immediately After Acute Myocardial Infarction Achieves Optimal Results

Cardiac rehabilitation is recommended in all patients with ACS, recent myocardial revascularization, stable angina pectoris, and stable coronary artery disease (CAD) (Taylor et al., 2022). Cardiac rehabilitation reduces risk factors, and increases the aerobic fitness, medication adherence, and survival after percutaneous coronary intervention and coronary artery bypass graft surgery (Colantonio et al., 2017; Rosenson et al., 2017). Moreover, it improves survival and reduces risk of recurrent MI in patients with acute MI (AMI) (Novaković et al., 2022). A previous meta-analysis comprising 63 trials and 14,486 patients assigned to exercise-based cardiac rehabilitation or no referral following MI or revascularization, reported that cardiac rehabilitation was associated with a lower risk of cardiovascular death [relative risk (RR) 0.74, 95% CI 0.64–0.86] and hospital readmission (RR 0.82, 95% CI 0.70–0.96) at 12-months (Anderson et al., 2016).

The rehabilitation is conducted to play an important role in achieving positive outcomes. Exercise training interventions had significantly higher beneficial effects on left-ventricular remodelling when exercise training is initiated immediately after AMI (from 1 week). Notably, when exercise was delayed for more than a week, the patients required an additional month of training to achieve the same level of benefit on cardiac remodelling as those who began exercise immediately after AMI (Haykowsky et al., 2011). A randomised controlled trial (RCT) comprising patients with AMI reported that early training intervention (<2 weeks post-MI) significantly improved health-related quality of life and functional capacity (through 6-min walk test) compared with the control (only usual care) (Peixoto et al., 2015). A previous clinical analysis was conducted using regression model to explore the recovery time and health of patients. The results showed that each 1-day increase in cardiac rehabilitation wait time led to a 1% reduction in the likelihood of improvement across all fitness-related measures including exercise level and Dartmouth quality of life physical fitness scale (Fell et al., 2016). Further, the findings showed that a shorter waiting period for cardiac rehabilitation after clinical coronary revascularization increases the health benefits of patients with coronary heart disease, such as optimizing cardiopulmonary function (peak oxygen uptake VO2peak; β = −0.165, *p* < 0.001) (Marzolini et al., 2015). This implies that individuals with AMI should begin a cardiac rehabilitation exercise immediately after hospital discharge to minimise risks of recurrence of cardiac events, reduce mortality risk, and improve quality of life (Parker et al., 2011).

### 2.2 Modality of Exercise Training in Cardiac Rehabilitation After AMI

Aerobic exercise training (AET) is the main exercise type in cardiac rehabilitation. AET improves health-related quality of life and several physiological parameters including cardiorespiratory fitness, cardiac function, handgrip strength, and knee extension strength (Izawa et al., 2004). The Exercise in Left Ventricular Dysfunction (ELVD) trial evaluated efficacy of exercise in patients with a first Q wave MI and a left ventricular ejection fraction (LVEF) below 40% and the results indicated that AET improved the quality of life of patients (Giannuzzi et al., 1997). Previous findings showed that 6-months AET markedly increased aerobic fitness and LVEF which are independent predictors of mortality in CVD patients (Keteyian et al., 2008; Kalam et al., 2014).

Studies have reported resistance training (RT) and AET result in a similar reduction (10%) in mortality rate in MI mice (Barboza et al., 2016). To increase the additional benefits of RT for patients with CVD, including those with MI and AMI, such as improved glucose metabolism, body composition, bone mineral density, muscle strength, and endurance (Williams et al., 2007; Kirkman et al., 2022), RT is mostly recommended in combination with AET (Williams et al., 2007; Price et al., 2016; Ambrosetti et al., 2020). Several studies have explored the benefits of combination of RT with traditional AET. A previous RCT of 26 MI patients was conducted with patients randomly assigned to AET with high-intensity group or to combined AET and RT group. The results showed that LVEF (Farheen et al., 2019), peak V̇O_2_ and quality of life (Khalid et al., 2019) were significantly improved in the combined group compared with the values in the AET group. Moreover, a Cochrane meta-analysis comprising 10,794 CAD patients showed that combination of AET and RT was associated with a 13% and 26% reduction in all-cause and cardiovascular mortality, respectively, and a 31% reduction in hospital readmission (Heran et al., 2011).

### 2.3 Dose of Exercise in Cardiac Rehabilitation After AMI

High-intensity interval (HIIT) and moderate-intensity continuous (MICT) training are complementary training modalities recommended by most exercise prescription guidelines for CAD patients (Vanhees et al., 2012; Mezzani et al., 2013; Price et al., 2016). Several meta-analyses have demonstrated that HIIT has similar or even higher benefits compared with MICT in improving peak VO_2_ (Conraads et al., 2015; Gomes-Neto et al., 2017). A previous RCT (Boidin et al., 2019) compared the effect of a 12-weeks HIIT to MICT in post-ACS patients (<6 weeks, 89% with MI) on the risk factors for arrhythmic death (i.e., heart rate recovery, heart rate variability, occurrence of ventricular arrhythmias, and QT dispersion (Wei et al., 1995; Freeman et al., 2006). The findings demonstrated that the two training interventions had no effects on these risk factors for arrhythmic death. Notably, a higher volume of exercise with MICT is required to achieve the same degree of reduction in all-cause and cardiovascular mortality observed with HIIT (Eijsvogels et al., 2016).

These findings indicate that an exercise training program should include moderate-to-high intensity AET and RT to markedly improve survival and quality of life in patients following MI. HIIT is a promising modality of exercise, owing to the lower dose of training needed to achieve the same magnitude of benefit as AET. Although HIIT is considered safe and effective in low-risk post-acute coronary syndrome patients, further research should be conducted to determine the safety in patients with AMI patients.

## 3 Irisin: Relation With Cardiac Rehabilitation-Mediated Protection

The novel myokine, irisin is a peptide obtained from hydrolysis of the transmembrane protein fibronectin type III domain protein 5 (FNDC5). Irisin has been found in both mouse and human serum following hydrolysis of FNDC5, and can be secreted in multiple tissues (Huh et al., 2012). Studies report that irisin is highly expressed after exercise, with plasma irisin levels peaking at 6 h after exercise, and returning to pre-exercise levels within 24 h, thereby mediating the beneficial effect of exercise (Pang et al., 2018; Maak et al., 2021). The effects of irisin in different tissues is dependent on metabolic phenotype. For instance, irisin levels in adipose tissue are significantly higher following HIIT compared with the levels after MICT in rats with dysregulated metabolic profile. However, the profile of irisin in skeletal muscle is not different after HIIT or MICT intervention. This implies that HIIT has protective effects against obesity and promotes metabolic dysfunction-induced reductions in adipose irisin levels (Tine Kartinah et al., 2018).

### 3.1 Relationship Between Irisin and Acute and Chronic Physical Activity Levels

#### 3.1.1 In Animals

Animal experiments have been conducted in the last 5-years to evaluate the effects of exercise patterns on irisin concentrations (Table 1). The experimental animal models included healthy, obesity (most), diabetic, aging, Alzheimer’s, and AMI. Three studies (Bell et al., 2016; Pang et al., 2018; Nadermann and Volkoff, 2020a) focused on the impact of acute exercise, and the results showed that irisin acts as an acute exercise effector and high expression of irisin was mainly detected in blood or muscle. Further, the effector of chronic exercise training was irisin or FNDC5 (Zhang et al., 2017; Kazeminasab et al., 2018; Lourenco et al., 2019), both detected in most tissues and organs. Notably, the expression patterns of irisin or FNDC5 were independent of the type of exercise (MICT, AET, and RT) and intensity or duration of the exercise. Findings on the profile of FNDC5 after exercise are not consistent. Training leads to a higher concentration of FNDC5 in hippocampus of Alzheimer’s mice (Lourenco et al., 2019). In addition, training upregulates expression of FNDC5 at protein and/or mRNA level in bone or muscle tissue in obese or normal members (Reisi et al., 2016; Rocha-Rodrigues et al., 2016; Zhang et al., 2017). However, some studies report that exercise training only upregulated FNDC5 protein content in skeletal muscles, but not its mRNA expression (Kazeminasab et al., 2018).

**TABLE 1:** Study characteristics of animal experiments that explored the effects of exercise on circulating irisin concentrations.

Author (year)	Subjects	Test area	Irisin (FNDC5) level	Exercise mode
Seo D.Y. (2020) (Seo et al., 2020)	Rat, Mouse	Circulating, adipose FNDC5 protein	↑	MICT treadmill, 8 and 12 weeks
Khalafi M. (2020) (Khalafi et al., 2020)				
Tine Kartinah N (2018) (Tine Kartinah et al., 2018)				
Kazeminasab F. (2018) (Kazeminasab et al., 2018)				
Shirvani H. (2020) (Shirvani and Arabzadeh, 2020)	Rat	Circulating Hippocampal	↑	MICT running 6 and 8 weeks
Babaei, A. (2021) (Babaei et al., 2021)				
Siteneski A. (2020) (Siteneski et al., 2020)	Rat Mouse	*Hippocampus*	↑	MICT treadmill, speed increase, 4 weeks
Gruhn, K. (2021) (Gruhn et al., 2021)				
Siteneski A. (2020) (Siteneski et al., 2020)	Rat	Circulating	↑	LICT treadmill, speed increase, 4 weeks
Hassaan P.S (2019) (Hassaan et al., 2019)	Rat	Skeletal	↑	LICT, treadmill, 8 weeks
Khalafi M.(2020) (Khalafi et al., 2020)	Mouse Rat	Circulating, adipose	↑	HIIT treadmill, 8,10, and 12 weeks
Shirvani H (2019) (Shirvani and Rahmati-Ahmadabad, 2019)				
Amri J. (2019) (Amri et al., 2019)				
Tine Kartinah N (2018) (Tine Kartinah et al., 2018)				
Kubo H. (2019) (Kubo et al., 2019)	Mouse	Circulating	↑	HIIT treadmill, speed increase, 12 weeks
Shirvani H. (2020) (Shirvani and Arabzadeh, 2020)	Rat	Circulating	↑	HIIT running 8 weeks
Liu (2021) (Liu et al., 2021)	Rat	Biceps brachii and surrounding fatty tissue	↑	high-intensity interval static training, 8 weeks
Nadermann N. (2020) (Nadermann and Volkoff, 2020b)	Goldfish	Muscle	↑	High intensity acute exercise, swimming, 30 min
Pang (2018) (Pang et al., 2018)	Mouse	Circulating	↑	Moderate acute treadmill, 30–60 min
Cho, E (2021) (Cho et al., 2021)	Mouse	Soleus and gastrocnemius muscle	↑	Acute Swimming 90 min
Hegazy, M. A. (2022) (Hegazy et al., 2022)	Rat Mouse	Hippocampi, muscle FNDC5 mRNA, circulating	↑	Swimming 4, 5, 6, and 8 weeks
Lourenco M.V. (2019) (Lourenco et al., 2019)				
Schaalan M.F (2018) (Schaalan et al., 2018)				
Belviranli M. (2018) (Belviranlı and Okudan, 2018)	Mouse Rat	Cardiac and hepatic, circulating, brain, brown/white adipose tissue, kidney, and pancreas, bone (FNDC5/irisin protein, mRNA)	↑	Voluntary wheel 2, 6, and 12 weeks
Uysal N (2018) (Uysal et al., 2018)				
Zhang J. (2017) (Zhang et al., 2017)				
Li (2021) (Li et al., 2021a)	Rat Mouse	Circulating Soleus muscles Cardiac	↑	RT, climb ladder, 8 and 12 weeks
Tavassoli H.(2019) (Tavassoli et al., 2019)			↓	
Kim HJ (2017) (Kim and Song, 2017)			↑	
Zhao (2021) (Zhao et al., 2021)	Rat Mouse	Circulating	↑	endurance training, 8 and 10 weeks
Amri J. (2019) (Amri et al., 2019)				
Bastu. E (2017) (Bastu et al., 2018)				
Mazur-Bialy A.I.(2017) (Mazur-Bialy et al., 2017)	Mouse Ra	Circulating, skeletal muscle	↑	Moderate endurance, treadmill or voluntary wheel, 8 weeks
Li (2017) (Li et al., 2017)				
Zhu (2021) (Zhu et al., 2021)				
Guiford BL (2017) (Guilford et al., 2017)	Mouse	Muscle	↓	Endurance voluntary wheel, 4 weeks
Babaei P (2017) (Babaei et al., 2017)	Rat	Circulating	↑	MICT and endurance, treadmill 8 weeks

FNDC5, fibronectin type III domain protein 5; MICT, moderate-intensity continuous training; HIIT, high-intensity interval training; LICT, low intensity continuous training; RT, resistance training.

#### 3.1.2 Clinical Studies

The ABCD study conducted in a general population demonstrated that irisin concentration is positively correlated with daily levels of physical activity (Buscemi et al., 2018). In addition, irisin concentration was correlated with gender (Zügel et al., 2016). Studies report that resting irisin concentration is higher in females compared with the level in males (Anastasilakis et al., 2014). Only few studies have explored response of irisin levels in patients with metabolic diseases undertaking different types of exercise. Findings from small sample clinical study showed no difference in exercise-induced (including high-intensity interval exercise, continuous moderate-intensity exercise, and resistance exercise) circulating irisin levels between healthy individuals and subjects with metabolic syndrome. This finding implied that the beneficial effects of exercise on glucolipid metabolism in patients with metabolic syndrome may be partly achieved by upregulation of irisin expression (Huh et al., 2015).

Six RCTs conducted from 2016 to 2021 explored the effect of various exercise modes on irisin expression, mainly in age-related, metabolic disease obesity (Weber-Rajek et al., 2019), progressive multiple sclerosis (Briken et al., 2016), non-alcoholic fatty liver disease models (Jia et al., 2018), and healthy young individuals (Qiu et al., 2018). The findings from these studies are summarized in Table 2. The findings from RCT studies indicated that exercise significantly upregulated expression of irisin (Kim et al., 2015; Briken et al., 2016; Bonfante et al., 2017; Jia et al., 2018; Weber-Rajek et al., 2019). Although further studies are required to verify these findings, these effects are possibly independent of exercise mode and duration of exercise. Notably, RT and combined exercise (CT) showed a higher increase in irisin levels relative to the levels in the aerobic exercise group (Jia et al., 2018). Moreover, RT and CT significantly improved metabolism and anthropometric indexes compared with the control (Amanat et al., 2020). These findings further confirm that exercise upregulates irisin level, implying that irisin is an effector molecule of exercise.

**TABLE 2:** Characteristics of randomized-controlled trials that explored the effects of exercise on circulating irisin concentrations in adults.

Author (year)	Participants	Age means (SD), exercise/control	Exercise mode	Irisin level
Briken S (2016) (Briken et al., 2016)	Patients with progressive multiple sclerosis	49.9 (7.6)/50.4 (7.6)	End, Acute and Chronic, 9 weeks	No sig
Bonfante IL (2017) (Bonfante et al., 2017)	Obese men	49.1(5.46)/49.1(6.33)	RT and End, (55–85% peak V̇O_2_), 24 weeks	↑ (Avoid reducing)
Qiu (2018) (Qiu et al., 2018)	Healthy young adults	27.4 (3.8)/24.7 (2.5)	acute exercise 80% peak V̇O_2_, 50 min and exhaustion	↑
Jia (2018) (Jia et al., 2018)	Patients with non-alcoholic fatty liver disease	54.62 (7.54) of aerobic/55.18 (7.48) of resistance/54.24 (7.51) of control	AET and RT, moderate intensity, 6 month	↑
Weber-Rajek M (2019) (Weber-Rajek et al., 2019)	Overweight or Obese Elderly Women with Stress Urinary Incontinence	62.5 (IQR: 2.0)/67.0 (IQR:6.0)	Pelvic floor muscle training, 4 weeks	↑
Amanat S. (2020) (Amanat et al., 2020)	Overweight women with metabolic syndrome	54.5 (6.9)	AET, RT, and CT, 12 weeks	↑

RT, resistance training; End, endurance training; AET, aerobic training; CT, combined exercise; V̇O_2,_ oxygen uptake; IQR, interquartile range.

### 3.2 Relationship Between Irisin Concentration and Cardiovascular Disease

Findings from animal experiments indicate that irisin is highly secreted in the myocardium (Aydin et al., 2014). Reduction of irisin concentration following AMI was first explored through animal experiments, even within 2 h post-AMI (Kuloglu et al., 2014). The findings showed that low irisin content is correlated with high expression level of markers representing myocardial damage (such as troponin and creatine phosphokinase-myocardial band isoenzyme). Several studies report similar findings in human trials (Emanuele et al., 2014; Anastasilakis et al., 2017). A cross-sectional study reported that the level of serum irisin in patients with coronary artery disease (CAD) was significantly lower relative to the level in the control, indicating that it is a potential independent predictor for CAD (Deng, 2016). Moreover, findings from a non-randomized, interventional study showed a significant negative correlation between circulating irisin and the degree of stenosis (Anastasilakis et al., 2017).

Myocardial hypoxia occurs after infarction. Compensatory reduction of irisin level induces reduction in ATP utilization and improves energy supply of ischemic myocardium (Kuloglu et al., 2014). However, reduction in irisin levels is exacerbated by aggravation of myocardial ischemia and hypoxia due to significant loss in myocardium, which induces ventricular remodeling and ultimately leads to heart failure (Matsuo et al., 2015; Zhang et al., 2020a). It is therefore inferred that moderate supplementation with irisin may help to improve post-infarction cardiac function.

### 3.3 Irisin Plays a Protective Role in CVD

revious findings indicate that expression of irisin is downregulated in some metabolic diseases, such as diabetes (Du et al., 2016). Stimulation of irisin may be an important molecular mechanism of metformin, a conventional drug for diabetes (Li et al., 2015). Irisin plays an important role in reduction of CVD risk factors and maintenance of cardiac function (Polyzos et al., 2018; Calan and Demirpence, 2019). However, it has also been suggested that abnormally high values of circulating irisin may be associated with increased risk factors for cardiovascular disease (Calan and Demirpence, 2019) and may predict major adverse cardiovascular events in patients with acute coronary syndrome (ACS) (Aronis et al., 2015). In contrast, most recent animal studies and studies using myocardial cell culture reported that exogenous irisin intervention effectively improved vascular endothelial function and impaired cardiac function under pathological conditions. To compare the recent studies on myocardial protection with irisin, the author lists them in Table 3.

**TABLE 3:** Myocardial protective effect of irisin.

Author (year)	Irisin interventions	Subjects	Models	Effect
Pan, J. A. (2021) (Pan et al., 2021)	i.p. injection, 2 weeks	Male 5-weeks-old C57BL/6J mice	doxorubicin -induced cardiotoxicity	Improve endothelial dysfunction
Liu (2018) (Liu et al., 2018)	i.p. injection, 16 weeks pre-incubation, 8 h	Mice and HUVECs	Diabetic cardiomyopathy	
Yan (2022) (Yan et al., 2022)	i.p. injection, 5 times	Mouse and Rat	Ischemia- reperfusion	Improve myocardial ischemia and hypoxia injury and dysfunction
Fan (2020) (Fan et al., 2020)	Incubation,25 h	H9C2	Hypoxia/reoxygenation	
Xin (2020) (Xin and Lu, 2020)	Incubation i.p. injection, 2 weeks and incubation 24 h	Primary cardiomyocytes	Myocardial infarction	
Liao (2019) (Liao et al., 2019)		Male mice and HUVECs		
Zhao (2019) (Zhao et al., 2019)	Incubation 24 h	CD-1 mice and Nkx2.5 + CPCs		
Deng (2020) (Deng et al., 2020)	Incubation 48 h, overexpression	Fluc+–eGFP + transgenic mice and BM-MSCs		
Ouyang (2020) (Ouyang et al., 2020)	Injection and incubation	Mst1 transgenic mice and Primary cardiomyocytes	LPS-mediated septic cardiomyopathy
Li (2019) (Li et al., 2019)	Overexpression and incubation 48 h	Irisin-Tgmice, primary cardiomyocytes	TAC induced cardiac hypertrophy
Hu (2022) (Hu et al., 2022)	Overexpression and subcutaneously infused 14 day	Young mice and Neonatal rat cardiomyocytes	Aging induced cardiac hypertrophy
Islam, M. R. (2021) (Islam et al., 2021)	AAV8-irisin-FLAG injection, once	Genetic deletion of Fndc5/irisin mice	Ageing or Alzheimer’s disease	Improve neuroregulation
Bretland, K. A. (2021) (Bretland et al., 2021)	i.p. injection, 4 weeks	Age-related tauopathy		

I.P., intraperitoneal; HUVECs, human umbilical vein endothelial cells; CPC, cardiac progenitor cell; BM-MSCs, bone marrow mesenchymal stem cells; LPS, lipopolysaccharide; TAC, transverse aortic constriction; Tg, transgenic; Fndc5, fibronectin type III domain protein 5.

Administration of irisin (0.5 μg/g body weight/day) in models of endothelial structural and functional abnormalities significantly reduces atherosclerosis in apolipoprotein E-deficient mice by reducing levels of inflammation and apoptosis (Zhang et al., 2016). Moreover, acute intravenous injection of irisin (10 μg/kg) reduces blood pressure in spontaneously hypertensive rats by promoting NO production and endothelial NO synthase (eNOS) phosphorylation in endothelial cells (Fu et al., 2016). Furthermore, determination of endothelium-dependent vasodilation shows that intraperitoneal injection irisin (0.5 μg g^−1^·day^−1^) daily in the morning for 8 weeks improves impaired endothelial function of the aorta caused by obesity (Han et al., 2015). In addition, irisin treatment (microinjected into the nucleus ambiguous) promotes neuronal depolarization through the blood-brain barrier and reduces abnormal heart rate response through central cardiovascular regulation (Brailoiu et al., 2015).

Findings from animal and cell studies indicate that administration of exogenous irisin alleviates function injury of various organs such as the liver (Bi et al., 2019), intestines (Du et al., 2019), pulmonary (Chen et al., 2017), cerebral (Jin et al., 2019), kidney (Zhang et al., 2020b), and heart (Wang et al., 2017; Gul-Kahraman et al., 2019) caused by ischemia and reperfusion. Previous findings indicate that the protective effect of irisin is attributed to its anti-inflammatory effect (Matsuo et al., 2015; Deng et al., 2018; Xiong et al., 2018), anti-oxidative stress activity (Zhang et al., 2020c) and effect on reducing endothelial injury (Ye et al., 2018). Recent studies reported that irisin plays an important role in alleviating tissue fibrosis (Zhou et al., 2018; Chen et al., 2019), improving mitochondrial function (Xie et al., 2015; Bi et al., 2019) and promoting angiogenesis (Song et al., 2014; Wu et al., 2015). Further, treatment of in the MI model with irisin (intraperitoneal injection or extracellular incubation) significantly reduces the level of cardiomyocyte apoptosis and myocardial infarct size, as well as significantly improves mitochondrial function, thus promoting recovery of ventricular function (Zhao et al., 2016; Wang et al., 2017; Wang et al., 2018; Li et al., 2019; Liao et al., 2019; Zhao et al., 2019; Deng et al., 2020; Fan et al., 2020; Ouyang et al., 2020; Xin and Lu, 2020). NKX2.5 + CPCs isolated from mouse embryonic stem cells were pre-treated with irisin and implanted into a myocardial infarction tissue. The findings showed significant increase in cardiac remodeling in post-MI hearts treated with irisin compared with controls (NKX2.5 + CPCs without irisin) (Zhao et al., 2019). The cardiac function and the related pathological indicators of AMI in mice treated with or without irisin were compared and the results showed that irisin reduced the area of MI and improved the cardiac function by activating ERK signaling pathways and promoting angiogenesis (Liao et al., 2019). Exercise upregulates the low expression of cardiac irisin caused by MI and further improves impaired cardiac function and renal function in animal MI rehabilitation model (Wu et al., 2020; Li et al., 2021a).

## 4 Possible Mechanisms of the Role of Irisin in AMI Rehabilitation

A previous study reported that exogenous irisin had therapeutic potential for pathologies associated with inflammation, oxidative stress, and apoptosis (Askari et al., 2018), but its receptor had not been identified. Recent studies have reported that simultaneous knockdown of integrins αV and β5 significantly attenuates the antioxidant/nitrosative stress and anti-apoptotic effects of irisin on H9C2, while pretreatment of adipose tissue-derived mesenchymal stromal cells (ADSCs) with irisin can promote ischemic cardioprotective effects by binding to cardiac integrins αV/β5 to induce the release of chemokine CSF2 from ischemic hearts and promotes the cardiac homing of ADSCs. It is therefore speculated that integrin αV/β5 may mediate the cardioprotective effects of irisin. However, the specific mechanisms involved in cardioprotection by irisin and its associated receptors still need to be verified by numerous studies. Mechanisms of irisin in cardiac protection reported in the past 5 years are summarized in Table 4.

**TABLE 4:** Possible mechanisms of the protective effect of irisin against CVD.

Author (year)	Experiment model	Possible mechanisms/signalling pathways	Protective effect
Hu (2022) (Hu et al., 2022)	Aging-related cardiac dysfunction in mouse	↓Lysosomal degradation of GLP-1R and ↑AMPKα ↓NLRP3	Anti-inflammatory
Li (2021) (Li et al., 2021b)	Sepsis-induced cardiac dysfunction in mouse	↓TLR4 and NLRP3 inflammasome signalings, ↓IL-1β, TNF-α, and IL-6	
Ning (2021) (Ning et al., 2021)	MI hearts in mouse	↑Nrf2/HO-1 axis and ↓NF-κB signaling pathway	
Lin (2021) (Lin et al., 2021)	Diabetic cardiomyopathy mouse	↑Integrin αVβ5-AKT signaling	Anti-oxidative stress (inhibition of apoptosis)
		↓iNOS/NOX2	
Deng (2018) (Deng et al., 2018)	HUVECs in AGEs medium	↓ROS, MDA, IL-1β and IL-18	
Yan (2022) (Yan et al., 2022)	Myocardial I/R injury in mouse	↑Integrin αV/β5, Csf2rb, ERK1/2-SOD2	
Jiang (2021) (Jiang et al., 2021)	Lipopolysaccharide-stimulated cardiomyocytes	↓Bax, caspase-3 and Fundc1	
Zhang (2020) (Zhang et al., 2020c)	DOX-induced cardiotoxicity in mouse	↑AKT/GSK3β/FYN/Nrf2 pathway	
Fan (2020) (Fan et al., 2020)	Hypoxia-reoxygenation injury in hyperglycemia-treated cardiomyocytes	↑AMPK pathway (↓LDH release)	Maintain mitochondrial function/structure, Suppress mitochondrial apoptosis
Xin (2020) (Xin and Lu, 2020)	Infarcted hearts *in vivo* and hypoxia-treated cardiomyocytes *in vitro*	↑Opa1-induced mitophagy	
Li (2018) (Li et al., 2018)	TAC-induced myocardial hypertrophy in mouse	↑AMPK-ULK1	
He (2021) (He et al., 2021)	Radiation-induced heart disease in mouse	↑DRP1, PINK1 and LC3B	
Nazem (2018) (Nazem et al., 2018)	Fndc5 knockdown in mice embryonic stem cell	↑PGC1-α	
Zhang (2019) (Zhang et al., 2019)	HUVECs and HMEC-1 were treated with oxLDL, Matrigel plug angiogenesis assay and CAM model	↑AKT/mTOR/S6K1//Nrf2 pathway	Promotes Angiogenesis
Liao (2019) (Liao et al., 2019)	Acute MI mouse	↑ERK pathway	
Yan (2022) (Yan et al., 2022)	Myocardial I/R injury in mouse	↑Integrin αV/β5, Csf2rb, ERK1/2-ANGPTL4	
Pan (2021) (Pan et al., 2021)	Doxorubicin induced cardiotoxicity in mouse	↓ROS, EndMT and UCP2	Anti-fibrosis
		↓NF-κB-Snail pathway	
Lin (2021) (Lin et al., 2021)	Diabetic cardiomyopathy in mouse	↑integrin αVβ5-AKT pathway	
Liu (2018) (Liu et al., 2018)		↓EndMT	
		↓TGF-β/Smad signalling	
Chen (2019) (Chen et al., 2019)	Angiotensin II-related cardiac fibrosis in mouse	↓ROS/TGFβ1/Smad2/3 signaling	
		↓Nrf2	
Yu (2019) (Yu et al., 2019)	TAC - induced cardiac hypertrophy in mouse	↑AMPK-mTOR signaling	
Yue (2021) (Yue et al., 2021)		↓NLRP3-mediated pyroptosis	

MI, myocardial infarction; TLR4, toll-like receptor 4; GLP-1R, glucagon-like peptide-1 receptor; NLRP3, nucleotide-binding oligomerization domain (Nod)-like receptor protein 3; HO-1, heme oxygenase-1; HUVECs, human umbilical vein endothelial cells; AGEs, advanced glycation end products; TAC, transverse aortic constriction; CAM, chicken embryo membrane; AMPK, adenosine 5’-monophosphate-activated protein kinase; AKT, protein kinase B; Bax, bcl2-associated x; mTOR, mammalian target of rapamycin; IL-6, interleukin-6; Csf2rb, colony stimulating factor 2 receptor, beta; I/R, ischemia-reperfusion; Fundc1, FUN14 domain-containing protein 1; TNF-α, tumor necrosis factor-α; ICAM-1, intercellular cell adhesion molecule-1; VCAM-1, vascular cell adhesion protein 1; ROS, reactive oxygen species; iNOS, inducible nitric oxide synthase; NOX2, NADPH oxidase 2; MDA, malondialdehyde; IL-1β interleukin-1β IL-18, interleukin-18; SOD2, superoxide Dismutase-2; GSK3β glycogen synthase kinase-3β oxLDL, oxidized low-density lipoprotein; Nrf2, NF-E2-related factor; LDH, lactate dehydrogenase; OPA1,optic atrophy 1; ULK1, uncoordinated 51-like kinase 1; PINK1, PTEN induced putative kinase 1; Fndc5, fibronectin type III domain protein 5; PGC1‐α, peroxisome proliferator-activated receptor-γ coactivator-1 alpha; Drp1, dynamin-1-like protein; ERK, extracellular regulated protein kinases; ANGPTL4, angiopoietin-likeProtein4; EndMT, endothelial-to-mesenchymal transition; UCP2, uncoupling protein 2; HMEC, human microvascular endothelial cells; TGF-β,transforming growth factor-β; SMAD, small mothers against decapentaplegic.

### 4.1 Inflammation

Exercise-induced irisin expression is implicated in regulation of several cardiovascular and metabolic conditions, and the therapeutic effect is partly attributed to the anti-inflammatory effects of irisin (Díaz et al., 2018). Findings from a human study confirmed that exercise-induced high irisin secretion is implicated in reduction in arterial stiffness and improvement of endothelial function through activation of the arterial AMPK/Akt/eNOS pathway in obesity (Inoue et al., 2020). Further animal experiments showed that irisin treatment significantly alleviates endothelial dysfunction in diabetic mice and downregulates mRNA expression of macrophages and T lymphocytes in atherosclerotic plaques as well as expression of inflammatory cytokines (IL-6, TNF-α) in aortic tissue, which further abrogates development of atherosclerosis, and analysis showed that these anti-inflammatory effects are correlated with activation of the AMPK/PI3K/PKB/eNOS pathway by irisin (Lu et al., 2015). Moreover, exogenous irisin supplementation in animal models has a direct therapeutic effect on atherosclerotic diseases by suppressing ox-LDL-induced cell inflammation and apoptosis. This therapeutic effect is attributed to inhibition of ROS/p38MAPK/NF-κB signaling (Zhang et al., 2016), and/or ROS/NLRP3 inflammasome signaling (Deng et al., 2018).

### 4.2 Antioxidation (Inhibition of Necrosis and Apoptosis)

Irisin plays a protect role against cardiomyocytes and vascular endothelial cells by reducing oxidative stress through AMPK-PI3K-Akt-eNOS-ROS pathway (Askari et al., 2018). A study using myocardial ischemia-reperfusion mice model showed that irisin treatment significantly increased activity of antioxidant factors such as SOD-1 and p38 and markedly reduces of myocardial infarct size (Wang et al., 2017) Moreover, irisin overexpression or irisin treatment exhibited cardioprotective effect by inhibiting ROS and upregulating expression of antioxidant molecules such as GSH and total SOD in acute and chronic cardiotoxicity models. The results showed that therapeutic activity of irisin was mediated through the AKT/GSK3β/FYN/Nrf2 axis (Zhang et al., 2020c). A recent study revealed that aerobic exercise alleviated the levels of oxidative stress and apoptosis in Type II cardiorenal syndrome (CRS II) model, which was partially mediated by increase in irisin secretion (Wu et al., 2020).

### 4.3 Irisin Maintains Mitochondrial Function/Structure

Abnormal structure and function of mitochondria and the resulting energy metabolism disorder plays a key role in cellular energy stress and apoptosis (Ma et al., 2017). Studies report that the protective effect of irisin on the injured myocardium due to cardiotoxicity or abnormal oxygen supply is attributed to improved mitochondrial function, autophagy regulation, and reduced apoptosis (Zhao et al., 2016; Zhang et al., 2020c). Exercise-induced irisin activated mitophagy and reduced MI area in a MI mice model and exhibited protective effects against cardiac function (Li et al., 2021a). These findings indicate that mitophagy is a potential mechanism through which exercise rehabilitation alleviates infarction. This finding was confirmed in other ischemia/reperfusion injury models, whereby irisin treatment restored integrity of the structure of mitochondria (by suppressing the opening of mitochondrial permeability transition pore and mitochondrial swelling) and restored mitochondrial respiration function (Wang et al., 2017; Bi et al., 2020). Recent studies report exogenous irisin administration alleviates pressure overload-induced cardiac hypertrophy by activating ULK1 autophagy pathway, whereas endogenous irisin knockout disrupts mitochondrial homeostasis and significantly decreases cardiac differentiation in mouse embryonic stem cells (Li et al., 2018; Nazem et al., 2018).

### 4.4 Angiogenesis

A previous study treated human microvascular endothelial cells (HUVEC) and transgenic TG (fil1: GFP) zebrafish with human recombinant irisin. The findings showed that administration of exogenous irisin upregulated expression of MMP-2 and MMP-9 (interstitial metalloproteinases) in vascular endothelial cells. This finding indicated that irisin modulates vascular growth of endothelial cells by regulates ERK pathway (Wu et al., 2015). Similarly, irisin could inhibit oxidized low-density lipoprotein (oxLDL) impaired angiogenesis by modulating ERK signaling pathways (Zhang et al., 2019). A recent animal study reported that administration of irisin after AMI reduces myocardial infarction size and improves cardiac function after MI (Liao et al., 2019). The therapeutic effect of irisin was attributed to the angiogenic effect mediated by HUVEC migration, which may be dependent on ERK pathway activation. However, studies have not fully explored the mechanisms underlying the effect of exercise-induced irisin vascular endothelial function.

### 4.5 Anti-fibrosis

The phenotype study found that irisin administration can significantly ameliorates fibrotic remodeling in post-MI hearts and alleviated injured cardiac function (Deng et al., 2020). Mechanistic studies reported that both myocardial FNDC5 overexpression and exogenous irisin administration attenuated cardiac adverse structural remodeling due to diabetes, including myocardial fibrosis, and that its protective effects were closely associated with activation of integrin αVβ5-AKT signaling and attenuation of oxidative/nitrosative stress (Lin et al., 2021). Further study found irisin treatment inhibited TGF‐β/Smad signaling and high glucose‐induced cardiac endothelial‐to‐mesenchymal transition (EndMT), which contribute to cardiac fibrosis and heart failure (Liu et al., 2018). It has also been suggested that this protection mechanism may be the result of Nrf2 mediated inhibition of oxidative stress in angiotensin Ⅱ related myocardial fibrosis model (Chen et al., 2019). Mice transverse aortic constriction (TAC)-induced cardiac hypertrophy model reported irisin treatment attenuates pressure overload-induced cardiac hypertrophy and fibrosis mainly through regulating AMPK-mTOR signaling or inhibiting NOD-like receptor protein 3 (NLRP3) -mediated pyroptosis activation (Yu et al., 2019; Yue et al., 2021). A recent study revealed that irisin as a mediator of the beneficial effects of exercise in cardioprotection like ameliorate EndMT through inhibiting activation of NF-κB-Snail pathway due to excessive accumulation of UCP2 and ROS and regulating the autophagy disorders (Pan et al., 2021).

In summary, these findings show that irisin (including exercise-induced irisin secretion) exerts a myocardial protective role through its anti-inflammation activity, antioxidant stress effect, and anti-apoptosis properties, as well as improving mitochondrial function, promoting angiogenesis and fibrotic remodeling. This indicates that irisin has high therapeutic and rehabilitation potential for treatment of patients post-MI. Further studies should explore the mechanism through which exercise prevents and alleviates heart disease and the role of irisin induced by exercise in these mechanisms.

## 5 Conclusion and Prospect

**FIGURE 1:**
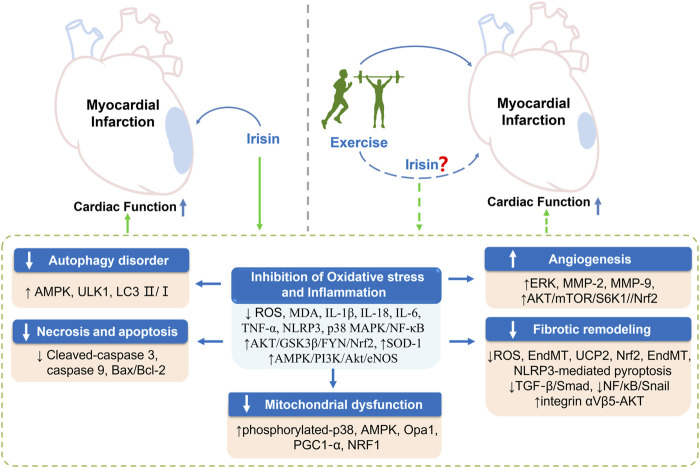
Exercise exhibits cardioprotective effect against post-myocardial infarction by mediating irisin expression and the potential mechanisms. Exercise and exogenous intervention with irisin can improve impaired cardiac function after MI by inhibiting inflammation and oxidative stress, and further improving the abnormalities of autophagy, apoptosis, and mitochondrial function, promoting angiogenesis, and inhibiting fibrotic remodeling caused by infarction. Meanwhile, exercise is an effective stimulus for upregulating irisin expression, however, whether exercise exerts the above beneficial effects through mediating irisin still needs to be verified by numerous studies. AMPK, adenosine 5′-monophosphate-activated protein kinase; PI3k, phosphoinositide 3-kinase; AKT, protein kinase B; eNOS, endothelial nitric oxide synthase; Bax, bcl2-associated x; mTOR, mammalian target of rapamycin; IL-1β, interleukin-1β; IL-18, interleukin-18; IL-6, interleukin-6; TNF-α, tumor necrosis factor-α; ROS, reactive oxygen species; MAPK, mitogen-activated protein kinase; NF-κB, nuclear factor kappa-B; NLRP3, nucleotide-binding oligomerization domain (Nod)-like receptor protein 3; MDA, malondialdehyde; SOD-1, superoxide Dismutase-1; GSK3β, glycogen synthase kinase-3β; Nrf2, NF-E2-related factor; OPA1, optic atrophy 1; ULK1, uncoordinated 51-like kinase 1; NRF1, nuclear respiratory factor; PGC1‐α, peroxisome proliferator-activated receptor-γ coactivator-1 alpha; ERK, extracellular regulated protein kinases; MMP-2, matrix metallo-proteinase-2; MMP-9, matrix metallo-proteinase-9; EndMT, endothelial-to-mesenchymal transition.

Exercise-based cardiac rehabilitation is an effective cardioprotective intervention strategy for patients with CVD (Anderson et al., 2016; Bennett et al., 2017). The protective effects of exercise on ischemic heart are partly mediated by vascular adaptations, mitochondrial biogenesis, as well as stimulation of skeletal and cardiac muscle tissue to release myokines including irisin (Fiuza-Luces et al., 2018). Irisin treatment improves outcomes of CVD, which is associated with its properties of reversing inflammation, oxidative stress and excessive apoptosis, implying that irisin is a promising therapeutic target for treatment of CVD. Notably, exercise can improve cardiac function following MI by upregulating myocardial irisin expression (irrespective of exercise mode). However, the exact mechanism has not been elucidated, and multiple clinical and animal studies should be conducted to explore the role of irisin in MI rehabilitation (Figure 1). In addition, studies should evaluate whether Irisin-related agents can be supplemented to improve clinical benefits in patients who are intolerant to exercise after MI. This review provides a possible reference for a therapeutic target for exercise rehabilitation in MI patients.
